# Effectiveness and Safety of Apixaban for Treatment of Venous Thromboembolism in Daily Practice

**DOI:** 10.1055/s-0040-1713683

**Published:** 2020-06-24

**Authors:** Stephan V. Hendriks, Frederikus A. Klok, Wilhelmina J.E. Stenger, Albert T.A. Mairuhu, Jeroen Eikenboom, Jaap Fogteloo, Menno V. Huisman

**Affiliations:** 1 Department of Thrombosis and Hemostasis, Leiden University Medical Center, Leiden, The Netherlands; 2 Department of Medicine, Haga Teaching Hospital, The Hague, The Netherlands; 3 Department of Internal Medicine, Leiden University Medical Center, Leiden, The Netherlands

**Keywords:** apixaban, direct oral anticoagulants, safety, efficacy, venous thromboembolism

## Abstract

**Introduction** Phase 3 trials have shown comparable efficacy of direct oral anticoagulants (DOACs) and vitamin K antagonists in patients with acute venous thromboembolism (VTE), with less major bleeding events in patients randomized to DOAC treatment. With DOACs being increasingly used in clinical practice, evaluation of the DOACs in daily practice-based conditions is needed to confirm their safety and effectiveness. The aim of this study is to evaluate the effectiveness and safety of apixaban in VTE patients in daily practice.

**Methods** In this retrospective cohort study, consecutive patients diagnosed with VTE in two Dutch hospitals (Leiden University Medical Center, Leiden and Haga Teaching Hospital, The Hague) were identified based on administrative codes. We assessed recurrent VTE, major bleeding and mortality during a 3-month follow-up period in those treated with apixaban.

**Results** Of 671 consecutive VTE patients treated with apixaban, 371 presented with acute pulmonary embolism (PE) and 300 patients with deep-vein thrombosis. During 3 months treatment, 2 patients had a recurrent VTE (0.3%; 95% confidence interval [CI]: 0.08–1.1), 12 patients had major bleeding (1.8%; 95% CI: 1.0–3.2), and 11 patients died (1.6%; 95% CI: 0.9–2.9), of which one patient with recurrent PE and one because of a intracerebral bleeding.

**Conclusion** In this daily practice-based cohort, apixaban yielded a low incidence of recurrent VTE, comparable to the phase 3 AMPLIFY study patients. The incidence of major bleeding was higher than in the AMPLIFY-study patients, reflecting the importance of daily practice evaluation and the fact that results from phase III clinical studies cannot be directly extrapolated toward daily practice.

## Introduction

Direct oral anticoagulants (DOACs) inhibit either thrombin (dabigatran) or activated factor X (apixaban, edoxaban, and rivaroxaban). Over the last years, DOACS are increasingly being used to prevent ischemic stroke in patients with atrial fibrillation and to treat acute venous thromboembolism (VTE). According to international treatment guidelines, the use of DOACS is being preferred over vitamin-K antagonists (VKA) for these two indications. 1 2 3 4 In VTE treatment, phase 3 studies have shown comparable efficacy of DOACs and VKA, with a better bleeding profile. 5 6 7 8 9 10 Furthermore, at prolonged treatment after the initial 6 months, DOACs have proven to be superior to placebo or aspirin for secondary VTE prevention. 11 12

Importantly, as phase 3 trials dictate to have strict in- and exclusion criteria both efficacy and bleeding rates may be underestimated because patients at higher risk of bleeding are usually excluded. With DOACs being increasingly used in clinical practice, evaluation of the DOACs using practice-based data sources is needed to better delineate their effectiveness and safety. Such data focusing on safety of apixaban for treatment of VTE are scarce.

In this study, we evaluated the efficacy and safety of apixaban in patients with VTE treated in two hospitals in the Netherlands.

## Methods

### Design and Patients

In this retrospective cohort follow-up study, consecutive patients diagnosed with VTE between January 2016 and December 2018 in two Dutch hospitals (Leiden University Medical Center, Leiden and Haga Teaching Hospital, The Hague) were identified via the hospitals' administrative system. Patients were eligible for inclusion if they were 18 years or older and had established acute symptomatic or incidental pulmonary embolism (PE) involving subsegmental or more proximal pulmonary arteries confirmed by computed tomography pulmonary angiography (CTPA), or symptomatic or incidental deep-vein thrombosis (DVT) of the lower or upper extremities, involving the popliteal, femoral, iliac, subclavian, axillary or brachial vein or the inferior vena cava, diagnosed by compression ultrasound or CT venography, or by a positive signal on magnetic resonance direct thrombus imaging (DTI) indicative of fresh thrombus in the proximal veins of the leg. 13 14 15

Patients were included in this study when the physician had the intention to start with apixaban treatment. In the Leiden University Medical Center, the treatment protocol recommended patients to be treated with apixaban 10 mg twice daily for 1 week after which apixaban 5 mg twice a day was initiated. In the Haga Teaching Hospital, the treatment protocol recommended patients to be initially treated with approximately 1 week of therapeutic weight based low-molecular-weight heparin (LMWH) after which apixaban 5 mg twice daily was given. Protocol deviations in both hospitals were common, truly reflecting practice-based medicine. Thus, the decision which of the two treatment regimens was initiated, depended on the discretion of the treating physician.

Patients who completed at least 3 months of anticoagulant therapy or met a study end-point in that period were included in this current analysis. Follow-up data were retrieved from the patient chart. Due to the retrospective study design, the need for informed consent was waived by the institutional review boards of both hospitals.

### Aims and Outcomes

The primary aim of this study was to evaluate the efficacy and safety of apixaban in VTE patients in daily practice. The primary efficacy outcome was recurrent VTE and all-cause mortality during a 3-month follow-up period after index VTE. The primary safety outcome was the 3-month incidence of major bleeding.

Secondary outcomes in this study were (1) the reported side effects of apixaban as noted by the treating physician in the patient chart and (2) the primary outcomes in the first week of treatment.

### Definitions

Recurrent VTE was defined as a new intraluminal filling defect on computed tomographic pulmonary angiography, confirmation of a new PE at autopsy, or a new intraluminal filling defect on computed tomographic angiography in other venous beds. Recurrent lower extremity DVT was defined as new noncompressibility by ultrasonography or as an increase in vein diameter under maximal compression, as measured in the abnormal venous segment, indicating an increase in thrombus diameter (≥ 4 mm), or by a positive signal on magnetic resonance DTI indicative of fresh thrombus in the proximal veins of the leg. 13 14 15

Major bleeding was defined according to the International Society of Thrombosis and Haemostasis (ISTH) criteria as any bleeding resulting in death, symptomatic bleeding in a critical organ (intracranial, intraspinal, intraocular, retroperitoneal, intra-articular and pericardial bleeding and muscle bleeding resulting in compartment syndrome) or symptomatic bleeding resulting in a decrease in the hemoglobin concentration of at least 2 g/dL or resulting in the transfusion of at least two packs of red blood cells. 16

In case of death, information was obtained from the hospital records. VTE-related mortality was defined as death within 7 days of PE diagnosis, PE confirmed as cause of death during autopsy, or sudden unexpected death with no other explanation. All events were adjudicated by two independent experts who were unaware of the initial management decision. Any disagreement between the two independent experts was resolved by a third expert.

### Statistical Analysis

For the presentation of the baseline characteristics, categorical data are presented as percentages or as proportion and continuous variables as means with standard deviation (SD). The main outcomes of the study are expressed by frequency and proportion with corresponding 95% confidence interval (95% CI). All adverse events were included in the primary analysis. The secondary outcome-reported side effect is provided as frequencies and proportion. SPSS version 25.0.0 (SPSS, IBM, Armonk, NY) was used to perform all analyses.

## Results

### Study Patients

Between January 2016 and December 2018, 671 consecutive patients were diagnosed with VTE and treated with apixaban, of whom 300 (45%) had DVT and 371 (55%) had PE with or without DVT. The baseline demographic and clinical characteristics of all 671 patients are summarized in Table 1 . Their mean age was 60 years (SD: 16), 48% was female and 6.3% had active malignancy at time of diagnosis. The median weight in this cohort was 85 kg (SD: 18.6) with 84 patients (13%) having a weight above 100kg. Renal insufficiency (creatinine clearance < 50 mL/min) was present in 60 patients (8.9%). Thirteen patients had severe renal insufficiency, a creatinine clearance estimated glomerular filtration rate < 30 mL/min (1.9%). The vast majority of the patients (74%) were treated as outpatient after initial index VTE; this was 93% for those with DVT and 58% for those with PE with or without DVT. For the patients treated initially in hospital, the median admission duration was 5.0 days (interquartile range 7).

**Table 1:** Baseline characteristics of patients with VTE treated with apixaban

	*n* = 671
**Demographics**	
Age, mean (SD)	60 (16)
Male sex, no (%)	347 (51.7)
Weight in kg, mean (SD)	84.7 (18.6)
< 60 kg—no (%)	26 (3.9)
60–100 kg—no (%)	354 (53)
> 100 kg—no (%)	84 (13)
Missing—no (%)	207 (31)
Body mass index, mean (SD)	27.3 (5.1)
Missing—no (%)	276 (41)
Creatinine clearance—no (%)	
< 30 mL/min	13 (1.9)
30–50 mL/min	47 (7)
50–80 mL/min	239 (36)
> 80 mL/min	319 (48)
Missing—no (%)	53 (8)
**VTE risk factors**	
Previous venous thromboembolism—no (%)	145 (22)
COPD—no (%)	65 (9.7)
Heart failure—no (%)	21 (3.1)
Estrogen use—no (%)	67 (10)
Immobilization—no (%)	174 (26)
Active malignancy no.—no (%)	42 (6.3)
Recurrent or metastatic cancer—no (%)	21 (3.1)
**VTE presentation**	
Qualifying diagnosis of VTE—no (%)	
PE with or without DVT	371 (55)
DVT only	300 (45)
Incidental PE no. (%)	16 (2.4)
Extent of qualifying PE no. (%)	
Subsegmental	37/371 (10)
Segmental	162/371 (44)
Central	165/371 (44)
Could not be assessed	7/371 (2)
**Treatment**	
Outpatient treatment	496 (74)
Readmissions	121 (18)
Apixaban without prior anticoagulant treatment	348 (52)
Apixaban with prior LMWH usage	323 (48)

Abbreviation: COPD, chronic obstructive pulmonary disease; DVT, deep-vein thrombosis; LMWH, low-molecular-weight heparin; PE, pulmonary embolism; SD, standard deviation; VTE, venous thromboembolism.

### Outcomes

During 3 months follow-up, two patients experienced a recurrent VTE (0.30%; 95% CI: 0.08–1.1; Table 2 ). A 71-year-old patient had progressive iliac vein thrombosis, 3 days after diagnosis of a DVT of the femoral vein and start of apixaban in the presence of a myelodysplastic syndrome. Another 49-year-old patient was diagnosed with symptomatic segmental PE, 1 month after initial DVT diagnosis, in the presence of a progressive stage IV nonsmall cell lung carcinoma.

**Table 2:** VTE-related adverse events of patients treated with apixaban

	Number	Incidence	95% CI
1. Overall mortality	11	1.6	0.9–2.9
2. Major bleeding	12	1.8	1.0–3.2
3. Recurrent VTE	2	0.30	0.08–1.1

Abbreviations: VTE, venous thromboembolism, 95% CI, 95% confidence interval.

A total of 12 patients (1.8%; 95% CI: 1.0–3.2) experienced major bleeding. The details of the major bleeding, its management, and outcome are provided in Table 3 . Of the 12 major bleedings, three occurred during the first week, including two major bleedings during LMWH therapy. One possible intracranial bleeding under LMWH was fatal; another major bleeding occurred in the presence of thrombocytopenia (platelet count 23 × 10*9/L); three patients (25%) had a malignancy.

**Table 3:** Detailed information of major bleeding

Patient	Sex	Age	Initial event	Time to adverse event	Major bleeding specified	Management and outcome
No. 1	F	74	PE	2 days	Decrease in the hemoglobin concentration > 2 g/dL and requiring transfusion 3 days postoperatively after total knee replacement on operative site during LMWH treatment	Management: Conservative, LMWH treatment was continued twice daily in therapeutic dosage followed by apixaban Outcome: Resolved without sequalae
No. 2	F	83	PE	5 days	Small traumatic intracerebral bleeding after a fall in the first week with LMWH treatment	Management: anticoagulant treatment was ceased. Temporary administration of prophylactic dosed LMWH. Apixaban was started 7 days later Outcome: Resolved without sequalae
No. 3	M	61	PE	7 days	Gastrointestinal bleeding resulting in decrease in the hemoglobin concentration > 2 g/dL, colonoscopy showed post colon polypectomy bleeding. Received infusion of thrombolytic drugs because of high-risk PE in beginning of admission 7 days prior	Management: administration of three packed red blood cells and 2000 IU prothrombin complex concentrate. Apixaban was temporary stopped with temporary administration of prophylactic dosage LMWH. Apixaban was restarted after successful clip closure of the post polypectomy bleed Outcome: apixaban was restarted 2 days after bleeding, patient was discharged 3 days after bleeding
No. 4	M	69	PE	8 days	Macroscopic hematuria resulting in decrease in the hemoglobin concentration > 2 g/dL after Millin prostatectomy	Management was started with operative evacuation of clots and continuous irrigating of the bladder via an indwelling catheter. Apixaban was switched to LMWH in a lower therapeutic dosage. After 26 days apixaban was restarted in the outpatient clinic Outcome: Resolved without sequalae
No. 5.	M	71	DVT	14 days	Bleeding in pancreas from pancreatic pseudoaneurysm	Management: coiling, anticoagulation was temporary stopped, temporary prophylactic dosage of LMWH was administered Outcome: discharged 1 day after coiling with the restart of anticoagulant treatment
No. 6	F	46	DVT	21 days	Abnormal menstrual bleeding resulting in decrease in the hemoglobin concentration > 2 g/dL after stopping oral contraceptives	Management: oral contraceptives restarted, tranexamic acid was refused by patient Outcome: Resolved without sequalae, apixaban was continued during the complete follow-up
No. 7	F	37	PE	37 days	Abnormal menstrual bleeding resulting in decrease in the hemoglobin concentration > 2 g/dL	Management: administration of tranexamic acid and iron infusion. Due to extent of bleeding, embolization of the uterine artery was necessary Outcome: Resolved without sequalae, after three days of cessation on anticoagulants, therapeutic dosages of LMWH were administered for 2 months, after which apixaban was continued
No. 8	M	62	DVT	42 days	A decrease in the hemoglobin concentration > 2 g/dL requiring transfusion because of gastrointestinal bleeding on due to diffuse vulnerable mucous membrane seen on endoscopic examination, post allogenic bone marrow transplantation due to myelodysplastic syndrome. (platelet count 23 × 10*9/L)	Management: thrombocyte transfusion, start of proton pump inhibition intravenously Outcome: no gastrointestinal bleed was objectified after 3 days of conservative therapy; anticoagulant treatment was continued
No. 9	M	82	PE	55 days	Progressive subdural hematoma and progressive subdural hygroma (both present before apixaban was started)	Management: anticoagulation was discontinued indefinitely Outcome: after initial progression of subdural fluid collection resulting in unilateral paresis of the arm, dexamethasone was administered, resulting in partial clinical recovery and regression of the fluid collection on CT
No. 10	F	76	PE	56 days	Gastrointestinal bleeding resulting in decrease in the hemoglobin concentration > 2 g/dL and transfusion required: clinical diagnosis diverticular bleeding, endoscopic examination showed no focus	Management: administration of intravenous tranexamic acid and 3500 IU prothrombin complex concentrate, anticoagulation was temporary stopped Outcome: resolved without sequelae after an admission of 3 days; apixaban was restarted the day after discharge
No. 11	F	57	PE	75 days	Ruptured spleen in patients with diffuse large B cell lymphoma with splenic localizations. Also, a large amount of hemorrhagic pleural effusion was drained by thoracentesis	Management: anticoagulation was discontinued indefinitely Outcome: patient also received first line of therapy for DLBCL and was discharged after an admission of 45 days
No. 12	M	59	DVT	81 days	Possible intracerebral bleeding in presence of progressive esophageal cancer while treated with LMWH. Symptoms of headache, nausea, and vision loss were present. Patient refused further treatment and decided to receive end-of-life care at home	Management: palliative treatment Outcome: patient died 5 days later

Abbreviations: CT, computed tomography; DLBCL, diffuse large B cell lymphoma; F, female; IU, international units; LMWH, light-molecular-weight heparin; M, male.

Note: Time to adverse event: time from initial events (and subsequent start of anticoagulant therapy) until occurrence of major bleeding.

Eleven patients (1.6%; 95% CI: 0.9–2.9) died during the 3 months follow-up ( Table 4 ). One patient on apixaban died of the index PE within 24 hours of the initial PE diagnosis. Seven patients (64%) had active malignancy at time of death and all died after initiation of palliative care at home or hospice because of metastasized end-stage disease. One patient died due to a possible intracerebral bleed; apixaban was already stopped and LMWH had been started.

**Table 4:** Detailed information of deaths

Patient	Sex	Age	Time to event	Specified
No. 1	F	93	0 days	Patient presented at ER with stridor and hypoxia. CT showed an incidental subsegmental PE. One single administration of apixaban was ordered. She died several hours after presentation with stridor, severe hypoxia, and laryngeal spasms. At autopsy, no good explanation was found for the upper airway narrowing as cause of death
No. 2	F	81	1 day	Patient using apixaban died of fatal PE, occurring 1 day after initial PE diagnosis with symptoms of progressive oxygen requirement and signs of exhaustion. Resection of a meningioma was the initial reason for admission, which was complicated by a pneumonia and acute PE. Due to severe comorbidity, that is, advanced age with frailty, severe emphysema, and a refractory delirium, palliative treatment was started
No. 3	M	87	10 days	Patient died due to progressive cerebral ischemia; on admission also an incidental segmental PE was diagnosed. Due to neurological deterioration and advanced age, a palliative treatment was started
No. 4	M	46	14 days	Patient was diagnosed with incidental PE in presence of a progressive stage IV NSCLC with obstruction of the right upper lobe bronchus, lymphangitis carcinomatosis, and pleural fluid. One day after initiation of palliative treatment, patient died
No. 5.	M	71	26 days	Patient died in a nursing home after neurologic deterioration due to progressive hydrocephalus. Initial admission was because of a subarachnoid bleeding treated with coiling of its aneurysm and extraventricular drainage. During hospital admission PE was diagnosed. Palliative treatment was initiated after neurological deterioration
No. 6	M	49	46 days	Patient died at home after initiation of palliative treatment. Multiple cerebral ischemic events occurred in the presence of a progressive stadium IV NSCLC resulting in a severe thrombophilic condition. Patient was also diagnosed with recurrent VTE during the 3-month follow-up
No. 7	M	57	56 days	Palliative treatment was initiated after admission of a subtotal ileus in the presence of metastasized gastric cancer with peritonitis carcinomatosis. Care was provided by the general practitioner
No. 8	F	64	74 days	Died at home after initiation of palliative treatment due to advanced stage NSCLC with bone and myogenic metastasis with progressive pleural carcinomatosis
No. 9	M	62	83 days	Patient died because of infectious complications after a hematopoietic stem cell transplantation due to myelodysplastic syndrome. Patient was admitted because of respiratory insufficiency after an aspergillus pneumoniae. After almost 3 months of admission, patient died one day after initiation of palliative treatment
No. 10	M	59	86 days	Patient died due to a possible intracerebral bleed. Patient also mentioned in major bleeding section: No. 12. Symptoms of nausea, headache, and hemianopsia were reported at home in the presence of progressive esophageal carcinoma without further treatment option. Apixaban was already ceased and patient was treated with LMWH. Palliative care was initiated by the general physician
No.11	M	67	89 days	Patient died after initiation of palliative treatment after small bowel ileus in the presence of a metastasized urothelial carcinoma with peritonitis carcinomatosis

Abbreviations: CT, computed tomography; ER, emergency room; F, female; LMWH, light-molecular-weight heparin; M, male; NSCLC, nonsmall cell lung carcinoma; PE, pulmonary embolism.

### Secondary Outcomes

The most frequent reported side effects of apixaban were headache (2.5%) and abdominal discomfort (2.4%). The following less frequent side effects were reported by the treating physician: nausea (0.9%), rash/hypersensitivity (0.4%), itching (0.8%), hair loss (0.3%), paraesthesia (0.3%), and dizziness (0.3%; Table 5 ) causing switch to an alternative anticoagulant in 13% of all 53 patients with side effects. All adverse events within the first week of anticoagulant treatment strategies are provided in Table 6 .

**Table 5:** Other reported side effect of apixaban

	Frequency	Incidence
1. Headache	17	2.5
2. Nausea	6	0.89
3. Abdominal discomfort	16	2.4
4. Itching	5	0.75
5. Hypersensitivity/rash	3	0.45
6. Hair loss	2	0.30
7. Paresthesia	2	0.30
8. Dizziness	2	0.30

**Table 6:** VTE-related adverse events in the first week according to initial treatment strategy

	Direct apixaban	Initial treatment with LMWH
1. Overall mortality	2/348	0/323
2. Major bleeding	1/348	2/323
3. Recurrent VTE	1/348	0/323

Abbreviations LMWH, low-molecular-weight heparin; VTE, venous thromboembolism.

## Discussion

In this practice-based study, we observed a lower rate of recurrent VTE (0.3% during 3 months) in patients treated with apixaban than that observed in the phase 3 AMPLIFY clinical trial (2.3% during 6 months). In contrast, the incidence of major bleeding (1.8% during 3 months) was higher than in the apixaban-treated patients in the AMPLIFY study (0.6% during 6-month follow-up).

The low rate of recurrent VTE could be explained by the difference in the follow-up duration in the phase 3 AMPLIFY clinical trial, which was twice as long. Moreover, a considerable percentage of recurrent VTE was adjudicated as death for which PE could not be ruled out. We therefore think VTE recurrence rates in both studies are likely comparable. Overall, the baseline characteristics in our cohort were comparable to those of the AMPLIFY study except that the proportion of patients included with a DVT was higher in the AMPLIFY study compared with 45% in this cohort. Moreover, more than half (52%) of our patients started apixaban without prior anticoagulant treatment, while this rate was 13% in the AMPLIFY study patients. Notably, the proportion of patients with initial LMWH treatment decreased over time, as experience and knowledge with apixaban treatment increased during the observation period.

The most notable difference of this analysis compared with the AMPLIFY study was the incidence of major bleeding. Taking a closer look at the patients who experienced a major bleeding episode elucidates the difference between our practice-based study and the phase 3 AMPLIFY study. First of all, two patients suffered from a hematooncological disease, with one being shortly after a hematopoietic stem cell transplantation, at time of bleeding. Overall, three out of 12 patients (25%) who experienced major bleeding had an active malignancy. Treatment of cancer-associated VTE is not only challenging due to a higher risk of recurrent VTE and mortality but also because of higher incidences of major bleeding. 17 The added value of DOAC therapy in patients with cancer-associated thrombosis has already been established with the publication of the SELECT-D3 and Hokusai VTE cancer trials, with consideration for the risk of bleeding in certain tumor types (e.g., gastrointestinal, urogenital). 18 19 International guidelines currently advise to consider the use of DOACs in cancer-associated thrombosis with caveats for these gastrointestinal and urogenital tumors. 4 In this respect, the fact that DOACs were sometimes prescribed in patients with cancer-associated VTE in this cohort reflects anticoagulant therapy in current daily practice. Second, in two patients bleeding occurred shortly after intervention; one patient already had a subdural fluid collection and one patient experienced bleeding within a week after prior treatment of thrombolytic therapy. These patients would have been excluded in phase 3 trials as they dictate strict in- and exclusion criteria.

We observed two heavy menstrual bleedings in this cohort. Treatment with factor Xa inhibitors is indeed associated with an increased risk of abnormal uterine bleeding, particularly heavy menstrual bleeding in premenopausal women when compared with treatment with VKA. 20 21 22 23 24 The observation that these women were admitted because of heavy menstrual bleeding, although it was not specifically monitored in this cohort, underlines the relevance of monitoring and counseling the risk of heavy menstrual bleeding in premenopausal women after initiating DOAC therapy.

Interestingly, in the management of major bleeding, prothrombin complex concentrate (PCC) was only used twice in patients with gastrointestinal bleeding, while all other patients with major bleeding were treated conservatively by only stopping the apixaban. This observation that most major bleeding events were managed conservatively, without the use of PCC, was also observed in the Dresden NOAC registry (PCC administered in 6.7% of all major bleeding events). 25

Overall, the rate of major bleeding in our cohort is comparable to rates of other practice-based cohorts in current literature. A systematic review including five large observational cohorts showed a 0.6 to 3.6% 3 months major bleeding rate in patients treated with apixaban for acute VTE. 26 Same proportions of major bleeding associated with DOAC therapy (3.3% during a mean follow-up of 85 days) were observed in a large practice-based multicenter, population study, although most DOAC users in this study used rivaroxaban. 27

The main limitation is the presence of selection bias as we do not know in how many patients (and why) another anticoagulant strategy than apixaban was chosen. Of note, apixaban was the first choice in anticoagulant therapy in both hospital protocols for VTE management. Therefore, we consider our results representative for daily practice since patients from both an academic and a nonacademic teaching hospital were studied and we observed rates of adverse events and mortality comparable to the published literature. Two of the major bleedings occurred on LMWH treatment in the first week of anticoagulant treatment, while the treating physician continued with apixaban treatment after the initial LMWH course. According to the intention to treat principle, we included those adverse events in the final analysis, which may have led to an overestimation of the apixaban associated rate of major bleeding. Strengths include the completeness of follow-up and the lack of exclusion criteria compared with clinical trials. Moreover, all outcomes were adjudicated by independent experts and we could provide detailed data on management and outcome for each adverse event.

In conclusion, apixaban yielded a low incidence of recurrent VTE in our large practice-based patient cohort. The incidence of major bleeding was, however, higher than in the AMPLIFY study, reflecting the importance of daily practice evaluation and the fact that results from phase III clinical studies cannot be directly extrapolated toward daily practice.
